# Curcumin and Quercetin as Potential Radioprotectors and/or Radiosensitizers for X-ray-based Sterilization of Male Navel Orangeworm Larvae

**DOI:** 10.1038/s41598-019-38769-3

**Published:** 2019-02-14

**Authors:** Pei-Shih Liang, Ronald P. Haff, Inna Ovchinnikova, Douglas M. Light, Noreen E. Mahoney, Jong H. Kim

**Affiliations:** United States Department of Agriculture – Agricultural Research Service – Western Regional Research Center, 800 Buchanan Street, 94710 Albany, CA USA

## Abstract

Two natural compounds (quercetin and curcumin) were tested as sensitizing or protecting agents for Navel Orangeworm (NOW) larvae under x-ray sterilization, with the aim to reduce required doses and thus facilitate the substitution of x-ray for radioisotopes. The compounds were added to NOW diet at concentrations between 0 and 1.0 mmol kg^−1^ and subsequent reared male larvae were subjected to x-ray irradiation (90 keV, 9 mA) to doses up to 15 Gy. Upon emergence as adults, surviving male NOW were paired with colony virgin females and placed in isolation for observation of deformity, mortality, and fertility. Treatments included rearing larvae on infused diet before irradiation, after irradiation, and both. Results were tabulated as percentage of insects that were dead/deformed, infertile, or fertile and subjected to chi-squared analysis. While insect populations subjected to quercetin treatments were not found to be significantly different from control at any x-ray dose, all curcumin treatments yielded significant differences at an absorbed dose of 10 Gy, both in terms of decreased mortality and fertility. While none of the treatments resulted in acceptable mortality/deformity rates, the observed effects strongly support the need for continued testing of natural compounds for their efficacy to reduce required dose levels for sterilization.

## Introduction

Navel orangeworm (NOW), *Amyelois transitella* (Walker) (Lepidoptera: Pyralidae), is the major pest of high-value California tree nuts (e.g. almonds, walnuts, and pistachios) and other specialty crops such as figs and oranges. NOW was first described in Arizona in 1922 where it was found on navel oranges and was first observed in California in 1942^1^. Past research has identified the insect as a secondary pest, occurring mainly on previously damaged or split nuts and fruits^2,3^. NOW larvae overwinter on “mummy” nuts which are mostly found on the orchard floor after harvest. Beyond direct losses from feeding damage, NOW is a significant food safety issue since the damage allows infestation by the mold *Aspergillus flavus* and subsequent aflatoxin contamination^4,5^.

Tree nuts are California’s largest agricultural product by value. In 2016, California pistachio and almond orchards covered approximately 1,180,000 acres, yielding 3.3 billion pounds of nuts valued at $6.5 billion. Furthermore, California produced 1.4 billion pounds of English walnuts valued at $1.2 billion in 2016 and the production has been steadily increasing for years^6^. The United States produces 80% of the world’s almonds, 24% of pistachios, and 29% of walnuts, almost all of which is grown in California.

Integrated Pest Management (IPM) strategies have been deployed to control NOW in California orchards for decades, comprised of traditional pesticides augmented by biological and cultural control strategies. Biological control includes predation (the introduction of natural predators that feed on NOW eggs) and mating disruption which employs synthetic pheromones (or other attractants) to interfere with the male’s ability to locate females in the field. Cultural controls include early harvest and postharvest sanitation (i.e. shaking unharvested and mummy nuts from the trees and removing them from the orchard floor). While these measures have been largely successful in reducing the levels of NOW damage, the past year (2017) has seen record damage as well as high levels of aflatoxin contamination due to extreme weather conditions and difficulties in implementing some of the control strategies^7^. Clearly, new and more reliable approaches are required.

The sterile insect technique (SIT) is an environmentally-friendly insect pest control method involving the mass-rearing of the target insect, radiation induced sexual sterility, and area-wide release of the sterile insect resulting in no offspring and a reduction of the wild population^8,9^. SIT was first employed in the United States during the 1950’s to eradicate the screw-worm, *Cochliomyia hominivorax* (Coq.)^10^. Integrated as one element with other suppression methods in area-wide IPM projects, the SIT has been successful in controlling a number of insect pests, including fruit flies (Mediterranean fruit fly, Mexican fruit fly, oriental fruit fly, melon fly), tsetse fly, and moths (codling moth, pink bollworm, false codling moth, cactus moth, and the Australian painted apple moth)^8,9^. To date, however, SIT has never been part of IPM strategies for controlling NOW, and literature reporting studies for NOW sterilization are very limited^2,11^. The Animal Plant Health Inspection Service (APHIS) has begun mass rearing and sterilizing NOW using gamma irradiation at their former Pink Bollworm (recently declared eradicated in the United States) Rearing Facility in Phoenix, AZ, and they report 400 Gy as the required sterilization dose for adult moths. Husseiney *et al*.^2^ reported sterilization doses for NOW using gamma irradiation for all life stages, and also indicated that adult males require approximately 500 Gy, while larvae require a much lower dose of somewhere between 30 Gy and 60 Gy. The study also indicated that for larvae and pupae, mortality rates were high at sterilization doses, indicating poor suitability for these life stages for SIT. This is likely due to complications with metamorphosis that are avoided when irradiating adults.

In almost all SIT programs, insect sterility is induced using gamma radiation from isotopic sources such as Caesium-137 or Cobalt-60. However, due to growing security difficulties associated with acquiring, maintaining, and transporting radioactive materials, the future availability and practicality of gamma irradiators is uncertain. Alternatives are urgently needed. X-rays are a logical choice for a variety of reasons. X-ray based irradiators only emit radiation when powered, generally require less shielding, and are subject to less stringent regulatory, security, and safety measures^12^. A small number of research studies investigating the use of x-ray irradiation to sterilize insect pests have been reported, including the Sweetpotato Vine Borer and Sweetpotato Weevil, the Mexican Leafroller, and the Light Brown Apple Moth^13–15^. Most studies indicate equivalent, if not better, results to that of gamma radiation in terms of induced sterility as well as subsequent fitness for release to compete with wild populations. Light *et al*.^11^ reported x-ray-based sterilization of adult male NOW at approximately 125 Gy as measured using a NIST calibrated ion probe, suggesting a fundamental difference in the biological response to x-rays vs. gamma.

There are a number of reasons why sterilization of NOW larvae would be advantageous over sterilizing adults. Larvae (or pupae) can be more easily contained for the irradiation procedure. In fact, adult moths must generally be chilled before irradiation to render them temporarily inactive to prevent mobility and escape. This increases the complexity and cost of the operation and has the potential for negative health impacts on the moths. For full-scale SIT programs, where millions of insects are reared and irradiated each day, the cost and reliability of currently available high-power x-ray units as well as the throughput of sterile insects are problematic. Recently, x-ray-based irradiators using commercially available, low energy soft x-rays have been developed^16^ and applied to the sterilization of NOW^11^. While these x-ray units have been shown to be robust, economical, and deliver a highly uniform dose if configured properly, they have not been shown to practically satisfy the required throughput for a large-scale SIT program. New approaches are required to increase throughput. Given the results of Husseiney *et al*.^2^, a logical approach would be to sterilize larvae, which require much lower doses. While gamma appears to induce unacceptable mortality at the required doses, the effect of x-ray at similar doses has not been tested. Additionally, reducing the required dose for insect sterilization has the potential to not only increase throughput, but also to reduce treatment costs, and negative health impacts on the target insect and thus increase their fitness (to compete with their wild counterparts) after release.

In biological systems, the co-application of certain natural compounds can enhance the effectiveness of traditional treatments through a mechanism termed sensitization. One example involves chemosensitization, in which the application of a sensitizing agent reverses resistance of fungal pathogens to commercial fungicides^17^. Natural compounds that pose no significant medical or environmental side effects are potential candidates for sensitizers in fungi^18^, but have not as yet been tested with insects. Ahmadi *et al*.^19^ showed that essential oils from medicinal plants work synergistically with gamma radiation to control the red flour beetle in stored products, but only in terms of lethality without consideration for the potential for sterilization. Hossain *et al*.^20^ also tested basil oil vapor and found it to have positive effects on radiation sensitivity in adult rice weevils, but again with the goal of reducing the required dose for lethality. While some antioxidant natural compounds may act as radiosensitizers, others may act as radioprotectors, alleviating the effects of ionizing radiation (i.e. gamma and x-rays), which interacts with biological molecules inducing free radicals that damage the organism^21^. This phenomenon has been reported in medicine for killing tumor cells while protecting healthy cells^22^ and destroying insects and/or bacteria on fruit with radiation while preserving or enhancing product quality^23^. It is therefore plausible that natural compounds may exist that sensitize insects in terms of required doses for sterilization while simultaneously reducing or eliminating normal side effects to radiation, yielding both greater throughput by reducing required dose as well as increased fitness of the sterilized insect. To our knowledge, studies on radioprotection and/or radiosensitization in terms of sterilizing insects for SIT have not been reported.

Flavonoids are among the most common groups of polyphenolic compounds in the human diet and are prevalent in plants. Flavonoids have been shown to have anti-inflammatory and antithrombic effects, protect low-density lipoproteins from oxidation, and promote relaxation of cardiovascular smooth muscle^24^. Past studies indicating potential synergistic effects when combining the flavonoid quercetin with chemotherapeutic agents or radiotherapy have been reported^25,26^. It has also been established that quercetin can act not only as a chemosensitizer and radiosensitizer but also as chemoprotector and radioprotector, protecting normal cells from the side effects that result from chemotherapy and radiotherapy^27^.

The Indian spice turmeric (*Curcuma longa*) has also been widely reported to offer many health benefits. Curcumin is the familiar name given to the most prevalent of the 3 curcuminoids that make up 4 to 5% of turmeric. Curcumin’s multiple immune-regulating benefits and anti-inflammatory characteristics are also well established. Studies have also shown that curcumin enhances chemotherapy and radiation treatments without generating toxic side effects and has potential application for mitigation of skin damage caused by radiation therapy^28,29^. Similar to quercetin, curcumin is considered as a radiosensitizer for various malignancies and a radioprotector for healthy tissue. Curcumin has been used as a radiosensitizer in cancer treatments including lymphoma, sarcoma, prostate, gynecologic, pancreas, liver, colorectal, breast, lung, and glioma^30^.

The objective of this study was to evaluate the natural compounds quercetin and curcumin as potential radiosensitizers and/or radioprotectors for NOW larvae sterilized using low energy (<100 keV) x-ray irradiation, with the goal of reducing required doses and/or increasing fitness of the resultant sterile moths. Reducing the required dose and/or increasing post-irradiation fitness would complement other efforts to develop x-ray as an alternative to gamma sources for large-scale SIT programs.

## Methods

### Insect rearing

A NOW colony was established in 2016 using eggs provided by Wonderful Orchards (Shafter, CA, USA). The eggs were disinfected for microbes by soaking for 15 min in a 0.02% benzalkonium chloride (Sciencelab.com, Inc., Houston, TX, USA) solution followed by 15 min continuous distilled water rinsing. Treated egg sheets were placed in one-gallon jars containing a previously reported red-flake organic wheat bran-based diet^11^. Hatched larvae were reared on the diet until adult emergence. The jar lids were lined with dimpled paper towels upon which adult females laid eggs, and upon completion of the life cycle these paper towels were used to propagate subsequent generations. The rearing room was maintained at 25–28 °C with a photoperiod of 12:12 (L:D) hr.

### Preparation of diet treatments

Quercetin (≥95%; Santa Cruz Biotechnology, Dallas, TX, USA) and curcumin (≥95%; Santa Cruz Biotechnology, Dallas, TX, USA) were added to NOW diet at various concentrations (Section 2.5) using alpha cellulose (Santa Cruz Biotechnology, Dallas, TX, USA) as a carrier. For 1.0 mmol kg^−1^ quercetin (MW = 302.2), 252 mg of quercetin was weighed into 25 ml volumetric flask and dissolved in acetone (Fisher Scientific, Waltham, MA, USA). Three ml of the solution was added to 2 g of alpha cellulose and stirred to evenly distribute the quercetin until the acetone was evaporated. The quercetin coated alpha cellulose was then incorporated in 100 g NOW diet by stir mixing. Other concentrations of quercetin incorporated diet were made from the dilutions of the 25 ml acetone solution following the same procedures. To generate 1.0 mmol kg^−1^ curcumin (MW = 368.4), 307 mg of curcumin was weighed into a 25 ml volumetric flask and dissolved in acetone. The same procedures were then followed to incorporate curcumin into 100 g NOW diet.

### Insect treatments

Third - fourth instar male larvae were hand-picked and placed in clear plastic 30-ml portion-cups with lids (four larvae per cup) containing treated or control diet. Larvae were left in the specific diet for various time periods before and/or after x-ray exposure (Section 2.4) as described in the experimental design (Section 2.5). As each test adult male emerged from pupation, it was paired with a virgin female of the same age (i.e. within one day of adult emergence) collected from the gallon colony jar and the pair was placed in a new 30-ml cup with regular diet and covered with dimpled paper towel for oviposition. To provide virgin female moths, rearing jars were emptied of moths in the morning, then in the afternoon as new moths emerged they were singularly transferred to individual clear 30-ml cups and their gender determined under a dissecting microscope. Paired moths remained together to mate until the end of their life cycle and the paper towels upon which eggs were laid were observed until any eggs hatched up to 3 weeks.

### X-ray irradiation

**Figure 1:**
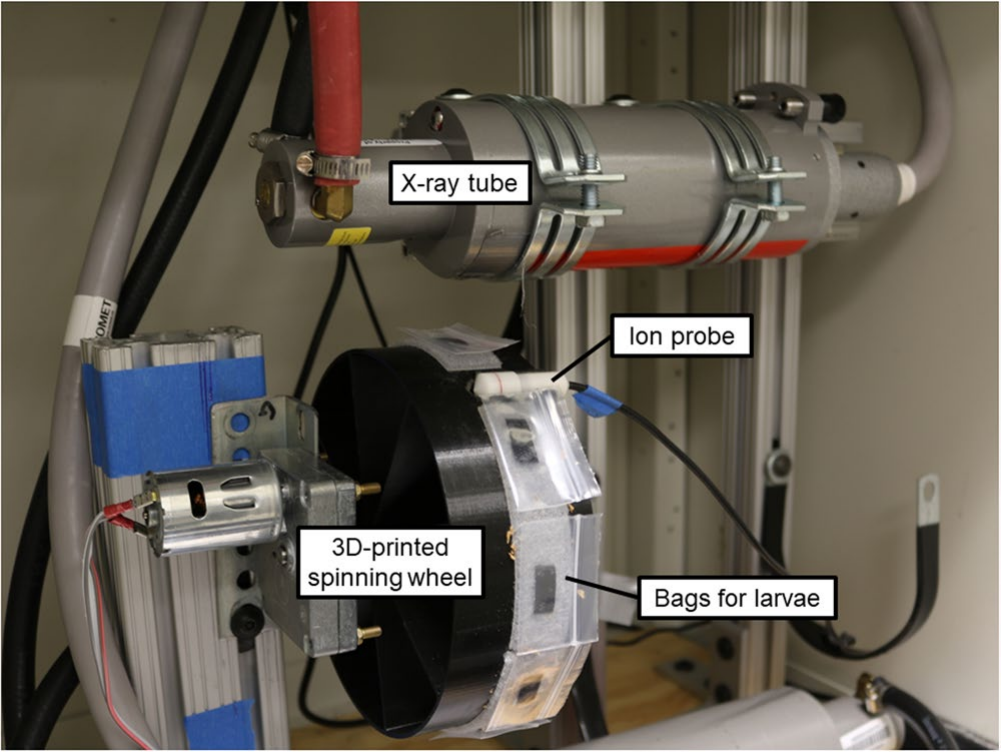
Configuration of the custom laboratory x-ray cabinet.

Irradiation treatments were performed in a custom laboratory x-ray cabinet (Fig. 1; CXR-105 x-ray tubes, Comet Technologies USA Inc., Stamford, CT, USA) at settings of 90 keV and 9 mA. The irradiator employed a spinning wheel positioned directly below the x-ray window. The wheel was sloped at an angle to the x-ray tube (to match the dose distribution emitting from the x-ray tube window) so that the x-ray dose horizontally across the surface of the wheel (left to right in Fig. 1) was uniform. Dose uniformity was established by trial and error (adjusting the angle) using x-ray sensitive film (Type HD-V2 Gafchromic dosimetry media, Ashland Corp., Bridgewater, NJ, USA) which was subsequently read with a radiochromatic optical density reader (Model 92D, Far West Technology, Goleta, CA, USA). Larvae were placed in one-inch plastic bags and attached to the wheel using Velcro. This configuration allowed the insects to travel in close proximity to the source for maximum dose. Since the larvae all travelled the same path below the x-ray window, each insect received a similar dose within a very small margin of error. Absorbed dose was measured with a radiation measurement system (Accu-Dose MNL/2086, Radcal Corporation, Monrovia, CA, USA) using a high dose rate ion chamber (10X6-0.18, Radcal Corporation). The wheel was custom built using a 3D printer (The Original Prusa i3 MK2, Prusa Research, Prague, Czech Republic) so that the ion probe snapped onto the wheel (but could simultaneously spin as the wheel rotated so that the feed wire did not tangle) and thus travelled the same path as the insects. The Accu-dose system was set in dose accumulation mode, so that the x-rays could simply be turned off when the desired dose was achieved.

### Experimental design

**Figure 2:**
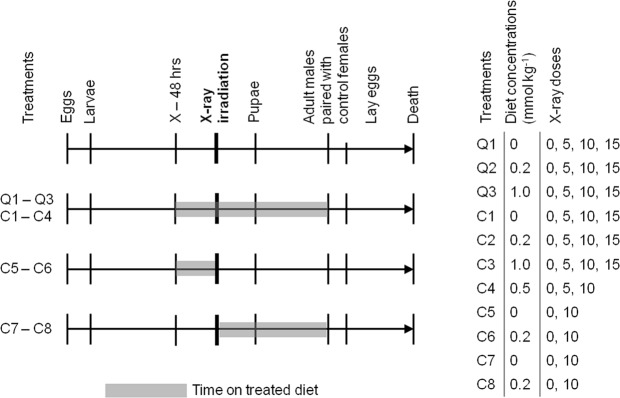
Experimental timelines for treatments.

Due to the colony size limitations and the number of available 3^rd^ and 4^th^ instar larvae at any given time, the experiments were performed in batches over the course of 6 months. Three sets of experiments were performed, and each subsequent set was modified based on results from its predecessors. The first set was conducted with larvae placed in quercetin treated diet at concentrations of 0 mmol kg^−1^ (control), 0.2 mmol kg^−1^ and 1.0 mmol kg^−1^ (treatments Q1–Q3). For each treatment, sub-treatments comprised x-ray irradiation to absorbed doses of 0, 5, 10, and 15 Gy. These values were selected based on preliminary experiments that indicate near 100% mortality at higher doses. Each diet treatment began 48 hours before irradiation, after which the insects remained on the treated diet until they emerged from pupation as adults. The second set of experiments was a repeat of the first, but with curcumin replacing quercetin (treatments C1–C3). Based on the observations for quercetin, an additional treatment at a concentration of 0.5 mmol kg^−1^ was added for curcumin while sub-treatment at 15 Gy was removed (treatment C4). In the final experimental set, larvae were placed on curcumin treatments either for only the 48 hours before irradiation then placed on control diet (treatments C5–C6), or only following irradiation (treatments C7–C8). Timelines for all treatments are shown in Fig. 2.

For each treatment, surviving and undeformed irradiated and control male moths were paired with virgin females. The behavior of the emergent adults and paired moths were then observed and classified in terms of the percentage of test larvae that: never emerged as adults or were deformed (dead/deformed), paired female yielded no eggs or whose eggs did not change from yellow to red (infertile), or paired female’s eggs turned red (fertile). Deformed males displayed a wide range of severity of deformation. For example, some were completely stuck in the cocoon and never emerged as functioning adults, while others managed to emerge but had asymmetric sized wings and could not fly properly.

### Statistical analysis

As described above, treatment results are recorded in terms of the percentage of samples that fall within each of three categories (dead/deformed, infertile, and fertile). Thus, the result variables are nominal (categorical) data. These results were analyzed using chi-squared test to determine whether mortality and fertility for the treated insects were significantly different from the untreated population (control). The chi-squared test is the nonparametric inferential procedure for testing whether the frequencies in each category in a sample represent certain frequencies in the population^31^. Data were grouped into two contingency tables; one for mortality (dead vs alive following irradiation) and one for fertility. For the fertility test, deformed insects were treated as a separate category of data. For the mortality tests, Fisher’s exact test was performed in addition to the chi-squared test, as is recommended in the case of relatively small number of samples. This was not possible for the fertility test since Fisher’s exact test requires a square contingency table. Statistical tests were performed using SigmaPlot12 (Systat Software Inc., San Jose, CA, USA).

## Results

### Quercetin treatments

Third to fourth instar male larvae were treated with quercetin incorporated diet at 0.2 mmol kg^−1^ (Q2), 1.0 mmol kg^−1^ (Q3), and control (Q1) starting from 48 hours before being irradiated at 0 Gy, 5 Gy, 10 Gy, and 15 Gy until adult emergence. There were 6–10 larvae in each treatment combination, depending on the number of larvae available in the colony at the time of preparation. Table 1 shows results in terms of the percentage of treated or untreated insects that were either dead/deformed, fertile, or infertile for each x-ray dose applied. Table 2 shows results in terms of the percentage of treated or untreated insects that were either dead/deformed or alive for each x-ray dose applied.

**Table 1:** Treatment effects on deformity and fertility of irradiated insects.

X-ray dose (Gy)	n	Control (%)			Treated (%)			χ^2^	p	Treatment
		Deformed	Infertile	Fertile	Deformed	Infertile	Fertile			
0	12	16.7	33.3	50.0	16.7	50.0	33.3	0.400	0.820	Q2 vs. Q1
5	15	22.2	33.3	44.4	16.7	16.7	66.7	0.760	0.680	Q2 vs. Q1
10	14	75.0	12.5	12.5	83.3	16.7	0	0.571	0.450	Q2 vs. Q1
15	15	88.9	11.1	0	83.3	16.7	0	—	—	Q2 vs. Q1
0	12	16.7	33.3	50.0	0	16.7	83.3	1.83	0.400	Q3 vs. Q1
5	15	22.2	33.3	44.4	50.0	16.7	33.3	1.32	0.520	Q3 vs. Q1
10	14	75.0	12.5	12.5	50.0	16.7	33.3	1.07	0.590	Q3 vs. Q1
15	16	88.9	11.1	0	85.7	14.3	0	—	—	Q3 vs. Q1

Deformed category includes dead insects. “n” Includes both Control and Treated. “−”: No data; χ^2^ cannot be computed with zeros in successive rows.

**Table 2:** Treatment effects on mortality of irradiated insects. Dead category includes deformed insects. Alive category corresponds to the sum of Fertile and Infertile from Table 1.

X-ray dose (Gy)	n	Control (%)		Treated (%)		χ^2^	p	p-exact	Treatment
		Dead	Alive	Dead	Alive				
0	12	16.7	83.3	16.7	83.3	0	1.00	1.00	Q2 vs. Q1
5	15	22.2	77.8	16.7	83.3	0.0700	0.790	1.00	Q2 vs. Q1
10	14	75.0	25.0	83.3	16.7	0.0800	0.778	1.00	Q2 vs. Q1
15	15	88.9	11.1	83.3	16.7	0.216	0.642	1.00	Q2 vs. Q1
0	12	16.7	83.3	0	100	0	1.00	1.00	Q3 vs. Q1
5	15	22.2	77.8	50.0	50.0	0.313	0.576	0.329	Q3 vs. Q1
10	14	75.0	25.0	50.0	50.0	0.162	0.687	0.580	Q3 vs. Q1
15	16	88.9	11.1	85.7	14.3	0.327	0.568	1.00	Q3 vs. Q1

“n” Includes both Control and Treated. “p-exact”: p-values from Fisher’s exact test.

### Curcumin treatments

The same experiment as described above for quercetin was conducted with curcumin added to the diet (treatments C1–C3). Resulting data was combined with the additional treatment of 0.5 mmol kg^−1^ diet (treatment C4), recalling that the 15 Gy exposure was omitted for 0.5 mmol kg^−1^ diet as described in the methods sections (Fig. 2). Thus, 3^rd^ to 4^th^ instar larvae were treated with curcumin incorporated diet at 0.2 mmol kg^−1^, 0.5 mmol kg^−1^, 1.0 mmol kg^−1^, or none (i.e. control) starting from 48 hours before x-ray irradiation at 0 Gy, 5 Gy, 10 Gy, and 15 Gy (with the exception of C4) until adult emergence. There were 6–30 larvae in each treatment combination. The results (treatments C2, C3, and C4) are shown in Table 3 for percentages of adult males that were dead/deformed, fertile, and infertile and Table 4 for mortality percentages.

**Table 3:** Treatment effects on deformity and fertility of irradiated insects.

X-ray dose (Gy)	n	Control (%)			Treated (%)			χ^2^	p	Treatment
		Deformed	Infertile	Fertile	Deformed	Infertile	Fertile			
0	63	10.0	20.0	70.0	9.10	21.2	69.7	0.0320	0.990	C2 vs. C1
5	46	19.0	23.8	57.1	4.00	28.0	68.0	2.67	0.260	C2 vs. C1
10	81	61.5	17.9	20.5	31.0	47.6	21.4	9.49	0.0870	C2 vs. C1
15	16	88.9	11.1	0	66.7	33.3	0	1.78	0.441	C2 vs. C1
0	42	10.0	20.0	70.0	8.30	16.7	75.0	0.105	0.949	C4 vs. C1
5	45	19.0	23.8	57.1	16.7	12.5	70.8	1.17	0.558	C4 vs. C1
10	59	61.5	17.9	20.5	30.0	25.0	45.0	5.66	0.0590	C4 vs. C1
0	47	10.0	20.0	70.0	0	11.8	88.2	2.60	0.272	C3 vs. C1
5	51	19.0	23.8	57.1	6.70	20.0	73.3	2.18	0.336	C3 vs. C1
10	75	61.5	17.9	20.5	30.8	38.5	30.8	6.17	0.0460^*^	C3 vs. C1
15	16	88.9	11.1	0	100	0	0	1.37	0.504	C3 vs. C1
0	62	10.0	20.0	70.0	12.5	15.6	71.9	0.260	0.878	C6 vs. C5
10	75	61.5	17.9	20.5	80.6	19.4	0	42.2	<0.001^*^	C6 vs. C5
0	62	10.0	20.0	70.0	15.6	28.1	56.3	1.27	0.530	C8 vs. C7
10	75	61.5	17.9	20.5	97.2	2.80	0	14.5	<0.001^*^	C8 vs. C7

Deformed category includes dead insects. “n” Includes both Control and Treated. Asterisks denote p-values that reject the null hypothesis at an alpha of 0.05.

**Table 4:** Treatment effects on mortality of irradiated insects. Dead category includes deformed insects.

X-ray dose (Gy)	n	Control (%)		Treated (%)		χ^2^	p	p-exact	Treatment
		Dead	Alive	Dead	Alive				
0	63	10.0	90.0	9.10	90.9	0.0940	0.759	1.00	C2 vs. C1
5	46	19.0	81.0	4.00	96.0	2.47	0.116	0.0600	C2 vs. C1
10	81	61.5	38.5	31.0	69.0	6.44	0.0110^*^	0.00800^*^	C2 vs. C1
15	16	88.9	11.1	66.7	33.3	0	1.00	1.00	C2 vs. C1
0	42	10.0	90.0	8.30	91.7	0.173	0.678	1.00	C4 vs. C1
5	45	19.0	81.0	16.7	83.3	0.0330	0.855	1.00	C4 vs. C1
10	59	61.5	38.5	30.0	70.0	4.07	0.0440^*^	0.0290^*^	C4 vs. C1
0	47	10.0	90.0	0	100	0.528	0.467	0.292	C3 vs. C1
5	51	19.0	81.0	6.70	93.3	0.826	0.363	0.214	C3 vs. C1
10	75	61.5	38.5	30.8	69.2	4.74	0.0290^*^	0.0230^*^	C3 vs. C1
15	16	88.9	11.1	100	0	0.152	0.696	0.500	C3 vs. C1
0	62	10.0	90.0	12.5	87.5	0.00800	0.928	1.00	C6 vs. C5
10	75	61.5	38.5	80.6	19.4	2.41	0.120	0.0820	C6 vs. C5
0	62	10.0	90.0	15.6	84.4	0.0790	0.779	1.00	C8 vs. C7
10	75	61.5	38.5	97.2	2.80	12.2	<0.001^*^	<0.001^*^	C8 vs. C7

Alive category corresponds to the sum of Fertile and Infertile from Table 3. “n” Includes both Control and Treated. “p-exact”: p-values from Fisher’s exact test. Asterisks denote p-values that reject the null hypothesis at an alpha of 0.05.

During the final experiments (treatments C6 and C8), curcumin incorporated diet was given to the larvae only before irradiation or only after irradiation. The aim was to determine whether curcumin acted to help protect the larvae from radiation (as a radioprotector) or to help recover from its damage (as an anti-inflammatory and antioxidant agent). To maximize the number of larvae per treatment, only 0 Gy and a 10 Gy x-ray dose were applied to larvae treated with 0.2 mmol kg^−1^ curcumin incorporated diet. For the combination of 0.2 mmol kg^−1^ with 0 or 10 Gy, there were 32–42 larvae in each treatment. The results are shown in Table 3 (deformity and fertility) and Table 4 (mortality).

## Discussion

### Quercetin treatments

The behavior of the control insects is roughly consistent with the findings of Husseiny^2^ with a few exceptions. Table 1 shows that at 15 Gy of exposure, the (surviving) moths are completely sterile with no red eggs observed for all treatments including control. However, in order to achieve that sterility, 89% of the control irradiated larvae were either dead or deformed and did not lay eggs in the first place. This behavior is consistent for the control in all treatments (Tables 1 and 2). This was basically as reported by Husseiny^2^, except the required doses are dramatically lower at 15 Gy for x-ray (at 90 keV) as compared to between 30 Gy and 60 Gy for gamma irradiation. This is consistent with observed values required for sterilization of adult moths, with reported values of 400 plus Gy for gamma vs. 125 Gy for 90 keV x-ray^11^. This suggests that either there is an error in dosimetry, or there is a fundamental difference in the biological effect of gamma vs x-ray irradiation. The dosimetry used for this study, as described earlier, is NIST certified and recently calibrated by the manufacture. Furthermore, ion probe dosimetry is considered the gold standard for measurements in these energy ranges. Therefore, it seems likely that the difference has to do with the interaction of the incident photons on the biological material. Further research is needed to explain this phenomenon, as it has far reaching consequences in the fields of food irradiation and phytosanitation, among many others.

At the 15 Gy level required for sterilization of diet treated larvae, quercetin results were somewhat ambiguous, with an improvement in emergence but also an increase in deformity rates. In terms of total red egg count, quercetin does not appear to have an effect, with the exception of the non-irradiated larvae in which case the percentage with red eggs rose from 50% for untreated to 83% for those treated at the 1.0 mmol kg^−1^ level (Table 1: Q3 vs. Q1). This suggests the possibility that quercetin may be desirable as a diet supplemental ingredient in colony rearing, yielding a higher viable egg count. This in turn may suggest greater overall colony health, regardless of irradiation status. This should not be surprising, given the known health benefits of quercetin and other antioxidant - anti-inflamatory compounds for various animals and humans. However, chi-squared analysis of the results failed to reject the null hypothesis that in terms of fertility the treated populations were the same as the non-irradiated control population at any dose level. In other words, the observed differences between the control and treated populations were not statistically significant.

### Curcumin treatments

#### Treatments applied before and after radiation (C2, C4, C3)

For controls at 15 Gy without treated diet, 88.9% of the larvae either died or were severely deformed (Tables 3 and 4). Among the remaining healthy adults, all produced yellow (infertile) eggs, but none produced red eggs. This suggests successful sterilization but suffers from unacceptably high mortality and deformity/disability. Even with curcumin incorporated diet, 15 Gy appears to be a supra-sterilizing dose with no red egg laying adults. At 10 Gy, mortality/deformity rates decreased, the rate of yellow eggs increased, and the rate of red eggs was unchanged or increased for all concentrations as compared to the untreated population. At 5 Gy of irradiation the larvae experienced fairly low death and deformity rates along with high red egg rates regardless of diet concentration. For non-irradiated control, the occurrence of red eggs increased as the concentration of curcumin increased, potentially indicating some positive gamete protection and health effects of curcumin. For doses of 0, 5, and 15 Gy, chi-squared analysis did not indicate significant differences between the treated and untreated populations. However, for irradiation doses of 10 Gy significant differences were found between treated and untreated populations at all diet concentrations of curcumin as indicated in Tables 3 and 4, both in terms of decreased fertility and decreased mortality. A dose of 10 Gy in combination with 0.2 mmol kg^−1^ curcumin diet resulted in the highest percentage of sterile adults (47.6% infertile). When higher concentrations of curcumin diet (i.e. 0.5 mmol kg^−1^ and 1.0 mmol kg^−1^) were applied to non-irradiated larvae, the ratio of fertile to non-fertile adults increased, indicating the benefits of curcumin infused diet for insect rearing with higher fertility overall.

#### Treatments applied only before (C6) or after (C8) irradiation

Table 3 indicates that the 0.2 mmol kg^−1^ curcumin diet applied only before irradiation to a dose of 10 Gy increased the percentage of non-fertile yellow eggs while eliminating fertile red eggs. However, giving the diet only after irradiation did not appear to have this effect. Additionally, when curcumin incorporated diet was only given before or after irradiation, the death and deformed rates were dramatically increased as compared to control, suggesting an increased sensitivity that manifested itself in mortality rather than sterility. Chi-squared analysis indicated significant differences between the treated and control populations for both treatments as compared to control.

### General discussion

It should be noted that the chi-squared calculations applied to the data in this study are not always as robust as we would like due to relatively small sample numbers. This is particularly true for the experiments with quercetin, in which the n was just 12 for each treatment. This leads to the situation in which some or all of the cells in the contingency tables used to calculate the chi-squared statistic have expected frequency values less than five. This does not invalidate the results of the calculation but can lead to a loss of accuracy. This is partially addressed by also running the Fisher’s exact test, the results for which are included in the tables for those with square contingency tables. This was not the case for any of the curcumin experiments at the 10 Gy dose level, which were the only results that yielded statistically significant differences between treated and control populations and therefore should not impact the conclusions that are drawn from the results. Sample sizes are generally less than we would have liked due to the intensive labor required to generate samples and the unpredictability of the number of appropriate larvae (as well as virgin females for pairing) available for use at any time from the colony.

There are strong motivations to irradiate NOW larvae rather than adult moths for SIT. Larvae are easier to handle physically, and do not need to be chilled for irradiation. More importantly, though, larvae are sterilized at much lower doses, helping increase throughput so that the use of x-ray as an alternative to radioisotopes becomes more feasible. The results of this study substantiated that this is true for x-ray as well as gamma, and further showed that required x-ray doses are significantly less than those required for gamma. The methods investigated here, if successful, would be very easy to implement in an SIT program for NOW, since it requires only a simple modification to the insect diet. Of course, the developed diet would apply only to NOW, and there is no reason to expect it to be applicable to other insects. Therefore, the techniques described here would have to be separately developed for any particular insect of interest for an SIT program, and this would be labor intensive and expensive. Clearly the two natural compounds as applied in these experiments did not produce ideal results in terms of enhancing the sterilization and fitness outcome for SIT, which would have been the observed cessation of fertile red eggs at 15 Gy (or lower) combined with lower mortality and deformity rates. However, the results did suggest that curcumin and quercetin appear to have radiosensitizing and/or radioprotecting effects when incorporated into insect diet. Chi-squared analysis confirms significant differences between treated populations for all treatments at the 10 Gy level of irradiation, although high mortality rates do not support the use of any of the tested treatments for use in an SIT program. Thus, there is a need for additional research in this area. Even just considering these two compounds, there are many factors in terms of concentrations and application methods that need a more thorough testing. Furthermore, these are just two of numerous natural (or synthetic) compounds with anti-inflammatory and antioxidant properties that could be explored. Finally, many other potential insect treatments (such as modified atmospheres) could be relevant. Unfortunately, the testing is tedious and labor intensive in terms of producing and testing sufficient numbers of larvae at the required age and finding the appropriate treatment for SIT may require the commitment of significant resources. However, given the urgent need as described above, the authors assert that such a commitment is justified.

## Conclusion

Quercetin and curcumin were incorporated into NOW diet at concentrations of 0, 0.2, 0.5, and 1.0 mmol kg^−1^ to test the efficacy of these natural compounds for either sensitizing NOW larvae to low energy x-ray irradiation or protecting them from the normal adverse side effects of irradiation. Male larvae reared on the modified diets were subjected to x-ray irradiation (90 keV, 9 mA) to doses up of 0, 5, 10, or 15 Gy. This was motivated by the desire to reduce the required sterilization dose and thus increase the feasibility of using x-ray as an alternative to gamma sources for the irradiation procedure. Subsequently, emergent male adults were paired with colony virgin females and kept in individual sample containers for observation of deformity, mortality, and fertility. Treatments included rearing larvae on infused diet before irradiation, after irradiation, and both. Resulting percentages of insects that were dead/deformed, infertile, or fertile were subjected to chi-squared analysis. Insect populations on quercetin-infused diet were not found to be significantly different from populations on untreated diet at any x-ray dose, regardless of whether the insects were on the diet before irradiation, after irradiation, or both. On the other hand, all curcumin treatments yielded significant differences at an absorbed dose of 10 Gy, both in terms of decreased mortality and fertility. None of the treatments resulted in mortality/deformity rates that would be acceptable for an SIT program. Nevertheless, the observed effects strongly indicate the need for more research on other natural compounds and their efficacy to reduce required dose levels for sterilization and increase the fitness of sterile insects.

## Data Availability

All data generated and analyzed during this study are included in this published article. Observation notes are available from the corresponding author on reasonable request.
